# Marburg Virus VP35 Can Both Fully Coat the Backbone and Cap the Ends of dsRNA for Interferon Antagonism

**DOI:** 10.1371/journal.ppat.1002916

**Published:** 2012-09-13

**Authors:** Shridhar Bale, Jean-Philippe Julien, Zachary A. Bornholdt, Christopher R. Kimberlin, Peter Halfmann, Michelle A. Zandonatti, John Kunert, Gerard J. A. Kroon, Yoshihiro Kawaoka, Ian J. MacRae, Ian A. Wilson, Erica Ollmann Saphire

**Affiliations:** 1 Department of Immunology and Microbial Science, The Scripps Research Institute, La Jolla, California, United States of America; 2 Department of Molecular Biology, The Scripps Research Institute, La Jolla, California, United States of America; 3 Department of Pathobiological Sciences, School of Veterinary Medicine, University of Wisconsin, Madison, Wisconsin, United States of America; 4 Division of Virology, Department of Microbiology and Immunology, Institute of Medical Science, University of Tokyo, Tokyo, Japan; 5 International Research Center for Infectious Diseases, Institute of Medical Science, University of Tokyo, Tokyo, Japan; 6 The Skaggs Institute for Chemical Biology, The Scripps Research Institute, La Jolla, California, United States of America; Institut Pasteur, France

## Abstract

Filoviruses, including Marburg virus (MARV) and Ebola virus (EBOV), cause fatal hemorrhagic fever in humans and non-human primates. All filoviruses encode a unique multi-functional protein termed VP35. The C-terminal double-stranded (ds)RNA-binding domain (RBD) of VP35 has been implicated in interferon antagonism and immune evasion. Crystal structures of the VP35 RBD from two ebolaviruses have previously demonstrated that the viral protein caps the ends of dsRNA. However, it is not yet understood how the expanses of dsRNA backbone, between the ends, are masked from immune surveillance during filovirus infection. Here, we report the crystal structure of MARV VP35 RBD bound to dsRNA. In the crystal structure, molecules of dsRNA stack end-to-end to form a pseudo-continuous oligonucleotide. This oligonucleotide is continuously and completely coated along its sugar-phosphate backbone by the MARV VP35 RBD. Analysis of dsRNA binding by dot-blot and isothermal titration calorimetry reveals that multiple copies of MARV VP35 RBD can indeed bind the dsRNA sugar-phosphate backbone in a cooperative manner in solution. Further, MARV VP35 RBD can also cap the ends of the dsRNA in solution, although this arrangement was not captured in crystals. Together, these studies suggest that MARV VP35 can both coat the backbone and cap the ends, and that for MARV, coating of the dsRNA backbone may be an essential mechanism by which dsRNA is masked from backbone-sensing immune surveillance molecules.

## Author Summary

Filoviruses, Marburg virus and five ebolaviruses, cause severe hemorrhagic fever that is characterized by suppression of the innate immune system. Important to immunosuppression is the viral protein VP35, which binds to and masks double-stranded (ds)RNA, a key signature of virus infection that is recognized by host sentry proteins like RIG-I and MDA-5. Previous crystal structures of VP35 from two ebolaviruses showed it to form an asymmetric dimer to cap the ends of dsRNA molecules. However, the question remained whether VP35 could mask remaining lengths of dsRNA between the ends from immune surveillance. Here we present the crystal structure of the dsRNA-binding domain (RBD) of Marburg virus VP35, alone and in complex with dsRNA. This crystal structure presents a very different arrangement of VP35s on dsRNA. Rather than binding only the ends, the Marburg virus VP35s spiral around the dsRNA backbone, continuously coating it. Additional biochemical experiments indicate that this continuous coating occurs in solution, and that like the ebolaviruses, Marburg virus VP35 is also able to cap the dsRNA ends, even though this was not apparent in the crystal structure. Together, this work illustrates how Marburg virus VP35 prevents recognition of dsRNA by backbone-sensing immune sentry molecules and provides an additional avenue for antiviral development.

## Introduction

Marburg virus (MARV) is an enveloped virus that belongs to the family *Filoviridae* and has a non-segmented, single-stranded, negative-sense RNA genome. Within *filoviridae* are genus *marburgvirus* which incudes two viruses, Marburg virus (MARV) and Ravn virus (RAVV), and genus *ebolavirus* which includes five viruses, Ebola virus (EBOV, formerly known as *Zaire ebolavirus*), Reston virus (RESTV, formerly known as *Reston ebolavirus*), Sudan virus, Taï Forest virus and Bundibugyo virus [1]. Filoviruses cause a severe viral hemorrhagic fever (VHF) in humans and non-human primates [2]–[4]. Large outbreaks of Marburg VHF have occurred in recent years in Angola and Republic of Congo, with case fatality rates close to 90% in Angola [4]. In general, outbreaks of the pathogenic filoviruses occur with 20–90% lethality, depending on the viral species, and likely, on the strength of host innate immune responses against the invading pathogen [5]–[7]. Interestingly, RESTV is non-pathogenic to humans, but highly lethal to non-human primates, and has recently been discovered among herds of domesticated swine in the Phillipines [8]. The World Health Organization has classified the filoviruses as Risk Group 4 pathogens and the development of protective vaccines or therapeutics against these viruses is a high priority.

Double-stranded RNA (dsRNA) is a unique product of viral infection and a key pathogen-associated molecular pattern (PAMP). Detection of dsRNA by host immune pattern recognition receptors activates signaling cascades that lead to production of interferons (IFNs). Survival from a filovirus infection may be determined, at least in part, by the strength of such innate immune responses mounted early in infection [5], [9]. In response, multiple families of viruses, including filoviruses, have evolved different strategies to counteract recognition of dsRNA [10]–[14].

Filoviruses encode a viral protein termed VP35 which is important for nucleocapsid assembly, replication and transcription and also plays a critical role in host immunosuppression [15]. VP35 binds to and likely masks viral dsRNA from host factors, which leads to suppression of RNA silencing, inhibition of phosphorylation/activation of interferon regulatory factor 3 (IRF-3) and antagonism of the type I IFN response [16]–[20]. EBOV VP35 has a flexible N-terminal region (amino acids ∼1–90), a central coiled-coil oligomerization domain (∼91–130), a flexible linker region (∼131–210) and a C-terminal dsRNA-binding domain (∼211–340) [21], [22]. In EBOV, VP35 contains an 11-residue N-terminal extension. As a result, equivalent positions of the C-terminal dsRNA-binding domain (RBD) of EBOV are numbered 11 higher than the corresponding amino acids in RESTV and MARV. For example, Arg 312 in EBOV is equivalent to Arg 301 in either RESTV or MARV.

Crystal structures of the VP35 RBD are available for EBOV and RESTV. These structures reveal that the RBD contains two subdomains, one of which is α-helical and the other is primarily β-sheet [22]–[24]. Each of these subdomains contains a basic patch that is conserved across the filovirus family. The “first basic patch” is contained in the α-helical subdomain, while the “central basic patch” is contained in the β-sheet subdomain [15].

A 170-residue version of the RESTV VP35 RBD was crystallized in complex with an 18-base pair oligomer [22]. In this asymmetric unit, there are four copies of the VP35 RBD bound to each oligomer, with two RBDs bound to each end of the dsRNA. The RESTV complex confers an overall appearance of a barbell with two copies of the VP35 RBD at each end and bare dsRNA in between. A ∼125 residue version of the EBOV VP35 RBD was crystallized in complex with a shorter, 8-base pair dsRNA oligomer and, in the crystallographic asymmetric unit, two copies of VP35 bind each end of the oligomer [10]. The shorter length of the crystallized dsRNA confers an overall appearance of a square of VP35s bound around central nucleic acids. The additional residues in the RESTV structure constitute a linker between the N-terminus of the RBD and the putative coiled-coil domain of VP35. These 60 residues are disordered and a functional role for this site is not yet known.

Importantly, in both crystal structures, the pair of VP35 RBD monomers at each end together forms an asymmetric dimer, which binds to dsRNA using a bimodal strategy [10], [22]. One of the monomers, termed the “backbone-binding” VP35, binds both strands of the dsRNA backbone using residues in and around its central basic patch (EBOV/RESTV numbering: R305/294, S272/261, Q274/263, and I340/329). The other monomer, termed the “end-capping” VP35, binds bases at the end of the dsRNA, as well as a portion of end-proximal backbone. This end-capping VP35 uses a hydrophobic patch to bind the terminal nucleotides and uses residues located in its central basic patch to bind the end-proximal portion of dsRNA backbone. Residues of the central basic patch that are used to bind dsRNA in the end-capping interaction are different from residues of the central basic patch that are used to bind dsRNA in the backbone-binding interaction. Mutation of key residues involved in either these end-capping or backbone-binding interactions has been shown to abrogate binding of dsRNA and restore host IFN signaling [10].

The protein–protein dimer interface between the backbone-binding and end-capping monomers is formed by central basic patch residues R312/301, R322/311 and K339/328 of the backbone-binding VP35 and E262/251, E269/D258 and D271/260 of the end-capping VP35 (EBOV/RESTV numbering). Note that in the end-capping monomer, R312/301 and R322/311 bind dsRNA backbone while in the backbone-binding monomer, R312/301 and R322/311 form the dimer interface. Together, the pair masks the end of the dsRNA oligo and occupies the recognition site of host immune sensors like RIG-I, MDA5 and LGP2 [25]–[27].

A question remaining from these previous structures is whether VP35 only binds the ends and end-proximal backbone of dsRNA molecules or if it is also able to coat the possible expanses of dsRNA backbone between the ends. Note that the backbone of dsRNA is the target of other viral IFN antagonists, such as the NS1 protein of influenza virus, B2 of flock house virus and P19 of Tombusvirus, and is a key recognition site for the host immune sensors RIG-I and MDA-5 that bind to dsRNA in a length-dependent manner [11]–[13], [28], [29].

Here, we report crystal structures of the MARV VP35 RBD, unliganded and in complex with a 12-base pair (bp) palindromic dsRNA. The structures and accompanying dsRNA-binding analysis performed by dot-blot assays and isothermal titration calorimetry reveal that MARV VP35 RBD coats the sugar-phosphate backbone along the length of dsRNA in addition to capping at the terminus, suggesting an alternate mechanism of dsRNA masking for immunosuppression. In addition, we demonstrate that MARV VP35 is a weaker IFN antagonist than EBOV or RESTV VP35, possibly due to the lower affinity of the MARV VP35 RBD for dsRNA. Suppression of IFN responses by VP35 is likely just one factor in virulence, as MARV can be just as lethal as EBOV. Indeed, recent outbreaks of MARV have occurred with 80–90% lethality [4].

## Results

### Crystal structure of MARV VP35 RBD

**Figure 1:**
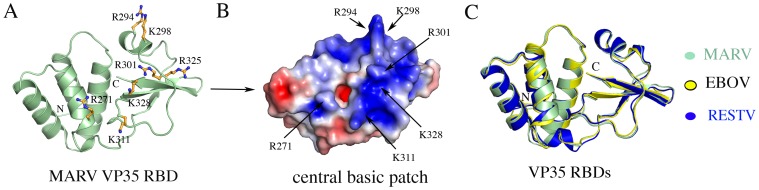
Structure of the MARV VP35 dsRNA binding domain (RBD). (A) Cartoon representation of the MARV VP35 RBD showing the α-helical and β-sheet subdomains. Basic residues in the central basic patch are shown in ball-and-stick representation and colored orange. (B) Electrostatic surface representation of MARV VP35 RBD with limits of ±3 *k_B_*T/e_c_ showing the highly conserved central basic patch. Key residues on the patch are labeled. (C) Superposition of VP35 RBD molecules from MARV (pale green), EBOV (yellow) and RESTV (dark blue) showing a conservation of the fold and secondary structure.

MARV VP35 RBD was crystallized in space group C222_1_ with one molecule of the VP35 RBD in the asymmetric unit (Table 1 and 2). Interpretable electron density was observed for residues 208–329 of the RBD. Like the VP35 RBDs of EBOV and RESTV, the RBD of MARV VP35 contains α-helical and β-sheet subdomains connected by a short loop (Fig. 1A). The β-sheet subdomain is formed by a three-stranded mixed β-sheet and an α-helix, and features the conserved, central basic patch critical to dsRNA binding, that contains residues R271, R294, K298, R301, K311, R325 and K328 (Fig. 1B). The α-helical subdomain contains a four-helix bundle followed by a short fifth helix and features the conserved first basic patch containing residues K211, K237 and K241 (Fig. S1).

**Table 1:** Data collection statistics.

	MARV VP35 RBD	MARV VP35 RBD+12-bp dsRNA
Wavelength (Å)	1.0000	1.0000
Space Group	C222_1_	P2_1_
a (Å)	42.52	36.59
b (Å)	90.88	99.19
c (Å)	65.92	81.80
Resolution (Å)	1.65	2.50
α (°)	90	90
β (°)	90	89.92
γ (°)	90	90
Total/Unique reflections	87222/15756	50876/20017
Redundancy	5.5(5.6)	2.5(2.5)
Completion (%)	100.0(100.0)	98.8(98.0)
I/σ	11.5(2.2)	8.5(2.2)
aR_sym_(%)	5.9(44.6)	13.1(36.2)
Matthews no. (Å^3^/Da)	2.4	2.8
Solvent content (%)	48.6	55.9

Values in parenthesis are for the highest resolution shell.

a R_sym_ = ΣΣ_i_|I_i_−<I>|/Σ<I>, where <I> is the mean intensity of the N reflections with intensities I_i_ and common indices h,k,l.

**Table 2:** Refinement statistics.

	MARV VP35 RBD	MARV VP35 RBD+12-bp dsRNA
Resolution (Å)	1.65	2.50
aR_cryst_	0.161	0.209
bR_free_	0.217	0.250
No of non-H atoms		
Protein	943	3772
RNA	-	504
Water	117	156
B-factors		
Protein (Å^2^)	23.8	16.4
RNA (Å^2^)	-	15.9
rms deviations		
bonds (Å)	0.006	0.001
Angles (°)	1.0	0.4
Dihedrals (°)	13.0	12.5
Ramachandran plot		
Most favored region (%)	97.1	97.4
Additional favored region (%)	2.9	2.6
Generously allowed region (%)	0	0
Disallowed region (%)	0	0

a R_cryst_ = Σ_hkl_||F_obs_|−k|F_cal_|/Σ_hkl_|F_obs_|, where F_obs_ and F_cal_ are observed and calculated structure factors, respectively.

b For R_free_, the sum is extended over a subset of reflections (∼5.0%) excluded from all stages of refinement.

The RBD of MARV VP35 is 43% and 41% identical in sequence to those of EBOV and RESTV, respectively, and the crystal structures align with a root mean square deviation (r.m.s.d.) of 1.07 Å and 0.92 Å, to EBOV and RESTV respectively (Fig. 1C). The location and in general, the contents of both the central and first basic patches are conserved among the structures.

### Complex of MARV VP35 RBD with dsRNA

MARV VP35 RBD was crystallized in complex with a 12-bp dsRNA oligonucleotide. Data were complete to 2.5 Å resolution and the structure was determined by molecular replacement in space group P2_1_ (Table 1 and 2). The asymmetric unit contains four molecules of the VP35 RBD bound to one molecule of 12-bp dsRNA. Interpretable electron density was observed for residues 208–329 of the RBD and all 24 bases of the dsRNA. Residues 205–207 are disordered in all four subunits. The MARV VP35 RBD protomers from the complex align with the unliganded MARV VP35 RBD with an r.m.s.d. of 0.44 Å suggesting no appreciable secondary structural changes occur upon dsRNA binding.

**Figure 2:**
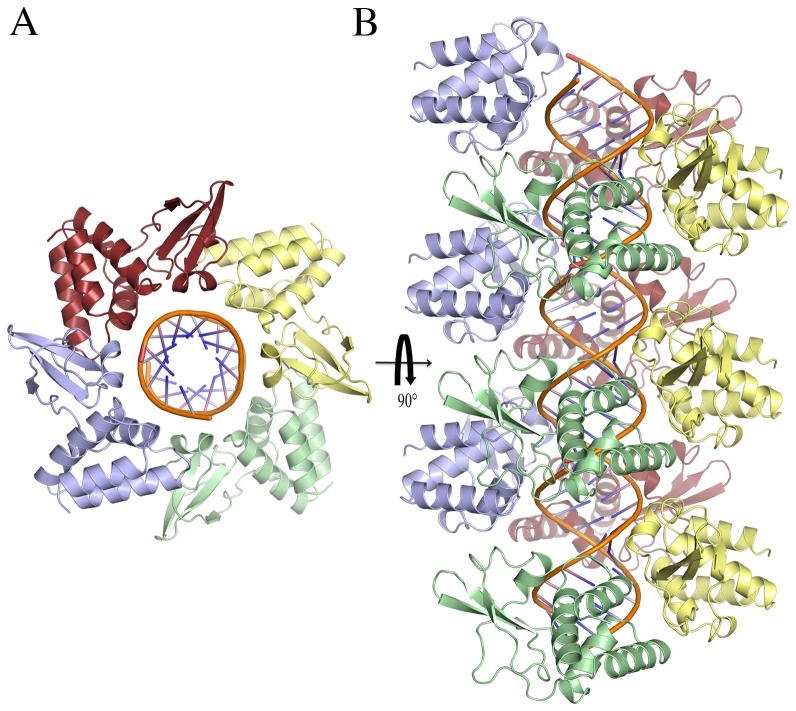
Structure of MARV VP35 in complex with 12-bp dsRNA. (A) Top view of the contents of the asymmetric unit. (B) Side view of the long pseudo-helical arrangement of dsRNA coated by MARV VP35 RBD, as formed by crystal packing. The four monomers in the asymmetric unit are colored pale blue, pale green, pale yellow and ruby, respectively.

In the previously reported EBOV and RESTV VP35 RBD structures bound to dsRNA, the ends of the dsRNA are bound by the VP35 RBD and do not interact with each other. By contrast, in the MARV VP35 complex structure, the terminal bases of each dsRNA in the asymmetric unit stack with those of the dsRNA in a neighboring asymmetric unit. Thus, in these crystals, the dsRNA molecules together assemble one, pseudo-continuous dsRNA helix (Fig. 2). The four MARV VP35 RBDs in the asymmetric unit do not end-cap, but instead, solely bind along the sugar-phosphate backbone, forming a continuous spiral of VP35 RBDs along the dsRNA. This arrangement results in a complete coating of the dsRNA by VP35 RBDs, with each of the conserved, central basic patches of VP35 RBD facing the dsRNA sugar-phosphate backbone (Figs. 2A and 2B).

**Figure 3:**
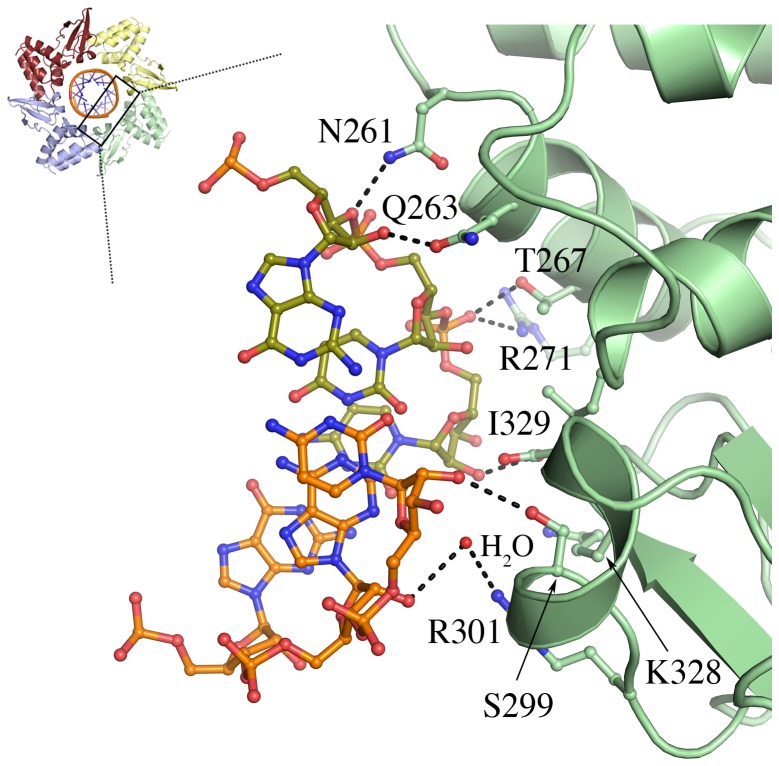
Key interactions of MARV VP35 RBD with dsRNA. dsRNA is shown in ball-and-stick representation. Hydrogen bonds are shown as black dotted lines. A bridging water molecule is shown as a red sphere. A representative MARV VP35 RBD is drawn in pale green.

Each VP35 RBD–dsRNA interaction involves ∼520 Å^2^ of buried surface and is accomplished by direct hydrogen bonds between N261, Q263, T267, R271, S299, and I329 and the ribose-phosphate dsRNA backbone (Fig. 3). N261 makes additional water-bridged hydrogen bonds to the dsRNA backbone, as do residues N224, R294, P295, R301 and K328. In addition, the side chain of K298 is positioned 4.5 Å from the backbone phosphate and may form favorable longer-range interactions. In EBOV and RESTV, the backbone-binding VP35 makes similar interactions with dsRNA although the identity of some of the residues differs by viral species.

Along the dsRNA, the α-helical subdomain of each bound RBD interacts with the β-sheet subdomain of a neighboring bound RBD, using an interface different from that observed between end-capping and backbone-binding VP35s in the RESTV and EBOV structures. For MARV, the neighbor interactions involve ∼340 Å^2^ of buried surface, a hydrogen bond between the amide nitrogen atom of A302 and the carbonyl oxygen atom of T219 and ∼20 non-bonded interactions (Fig. S2).

It is interesting that we observe no “end-capping” interactions in this MARV VP35 RBD - dsRNA complex. We performed biochemical experiments to determine whether the end-capping type of VP35 RBD binding could occur for the MARV VP35 RBD in solution, even if it did not appear in these crystals.

### dsRNA binding assays

**Figure 4:**
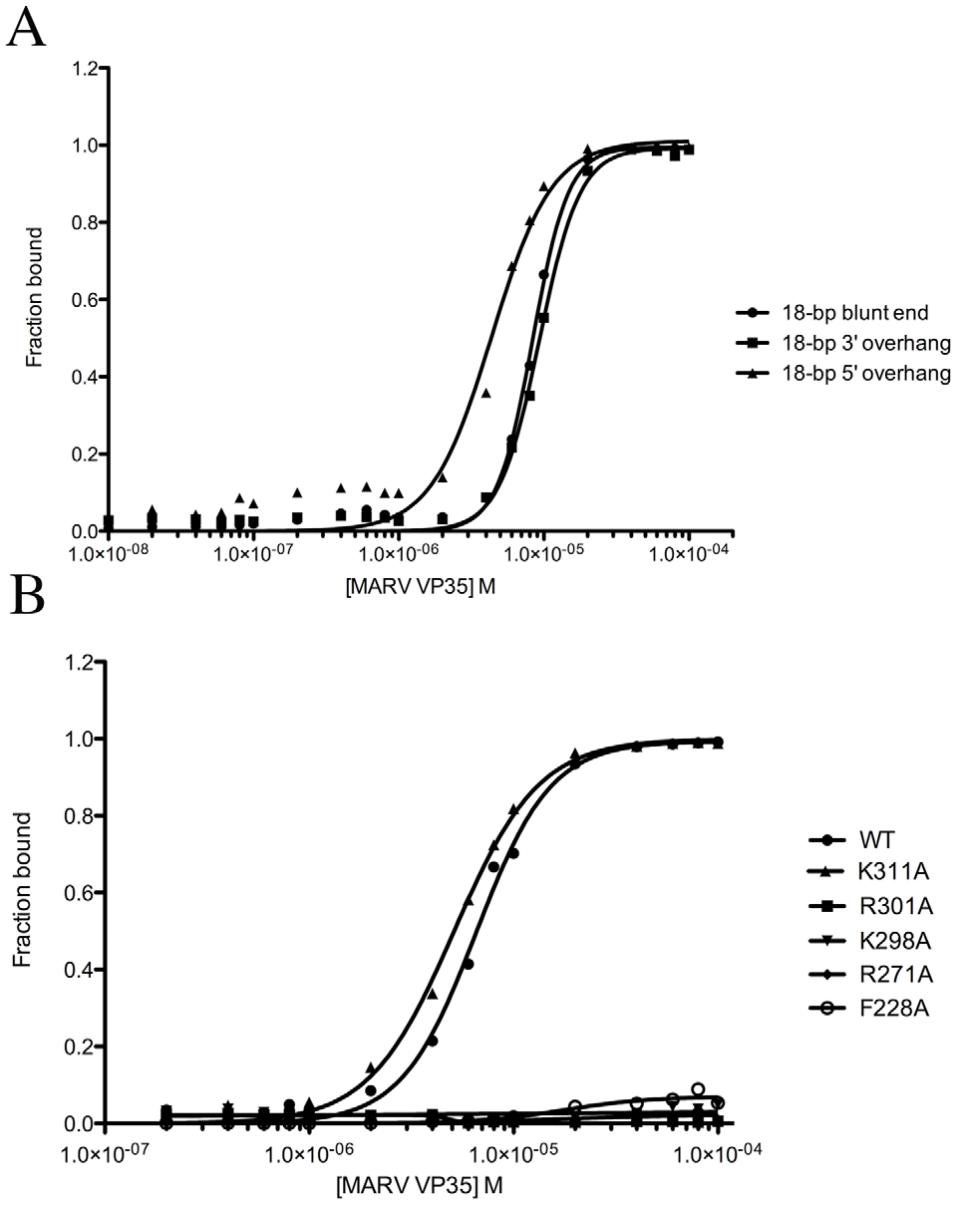
Binding of wild-type and mutant MARV VP35 RBD to dsRNA. (A) MARV VP35 binds with similar affinity to 18-bp dsRNA with blunt ends or with a 3′ or 5′- overhang. (B) Binding of R301A, K311A, K298A, R271A, and F228 mutants of MARV VP35 RBD to 18-bp blunt ended dsRNA. Only the wild-type VP35 and the K311A mutant bind to dsRNA.

Dual-filter dot-blot binding assays with radiolabeled dsRNA were used to investigate binding of MARV VP35 RBD to dsRNA. MARV VP35 RBD binds to 18-bp RNA with blunt ends or a 3′ or 5′ overhang with similar affinity, in a cooperative manner (Fig. 4A, and Table 3). Multiple residues were mutated separately to alanine and tested for binding to an 18-bp blunt-ended dsRNA oligomer. We find that R271A and R301A mutations (central basic patch residues that contact dsRNA) each disrupt binding to dsRNA. Interestingly, a K298A mutation, which is 4.5 Å from dsRNA phosphate backbone, also disrupts dsRNA binding (Fig. 4B). Thus, although it is not readily apparent in the crystal structure, the residue K298 appears to be important for dsRNA recognition and binding.

**Table 3:** Binding affinities and Hill coefficients of various VP35 proteins binding to dsRNA.

	MARV VP35 RBD		EBOV VP35 RBD		RESTV VP35 RBD	
18-bp dsRNA	K_d_ (µM)	Hill coeff.	K_d_ (µM)	Hill coeff.	K_d_ (µM)	Hill coeff.
Blunt-ended	8.4±0.2	3.5±0.3	1.9±0.1	2.9±0.3	2.5±0.1	2.6±0.2
3′ overhang	9.3±0.2	3.1±0.2	17.1±0.7	3.6±0.4	6.0±0.2	3.4±0.4
5′ overhang	4.4±0.3	2.1±0.2	ND	ND	ND	ND

Mutation of K311A (a central basic patch that does not contact dsRNA) has no effect on binding 18-bp dsRNA as one might expect (Fig. 4B). Interestingly, the corresponding residues of the end-capping monomers in RESTV (R311) and EBOV (R322) make hydrogen bonds to dsRNA, and those of the backbone-binding monomers in RESTV and EBOV form hydrogen bonds to E251 (E262 in EBOV) across the dimer interface. An R322A mutation in EBOV VP35 does abrogate binding to an 8-bp dsRNA [10].

In RESTV and EBOV, the protein carboxy terminus at residue I340/329 and the side chain of the penultimate residue K339/328 form critical hydrogen bonds to the dsRNA backbone [10], [22]. These particular contacts appear not to be critical for MARV, as MARV VP35 truncated at position 327 (MARV VP35_205–327_), immediately prior to these residues, binds to dsRNA with an affinity similar to that of wild-type (Fig. S3).

Additional dot-blot binding data suggest that filovirus VP35 RBDs bind blunt-ended dsRNA with µM affinity: ∼2 µM for EBOV, ∼2.5 µM for RESTV and ∼8.5 µM for MARV (Table 3, Fig. S4). Note that MARV VP35 RBD binds blunt-ended dsRNA with 3–5 fold lower affinity than the EBOV and RESTV VP35 RBD. The presence of a 5′ overhang on dsRNA increases the affinity of MARV VP35 RBD to dsRNA by 2-fold. The presence of a 3′ overhang only slightly diminishes affinity of MARV VP35 RBDs for dsRNA, but greatly diminishes binding of EBOV and RESTV VP35 RBDs (Table 3, Fig. 4 and S4).

### Hydrophobic residues in the end-capping monomer of ebolaviruses

In the crystal structures of the EBOV and RESTV VP35 RBDs bound to dsRNA, a key Phe of the end-capping molecules (F239 in EBOV and F228 in RESTV) makes hydrophobic interactions with neighboring residue I340 and also with the terminal base of dsRNA. It was thought that contact of this Phe to the RNA base could be important as an F239A mutation in EBOV abrogates binding to dsRNA [10]. However, in the backbone-binding copy of EBOV and RESTV VP35 and in all four MARV VP35s, F228/239 makes no contact to dsRNA. The backbone-binding VP35s would presumably bind dsRNA just as well if F228/239 were mutated, yet dsRNA binding is blocked. It is possible that the importance of this residue does not lie in a critical aromatic stacking interaction with dsRNA, but instead in maintenance of the VP35 structure. The residue F228 is located in a hydrophobic pocket that bridges the α-helical subdomain to the β-sheet subdomain. In addition, F228 is located adjacent to residues Q263 and T267 that make direct bonds to dsRNA and might play a role in positioning the residues for hydrogen bonds with dsRNA. Indeed, dot-blot binding assays reveal that while MARV F228A VP35 is unable to bind dsRNA, F228L binds dsRNA at wild-type levels (Figs. 4B, S3). 2D NOESY NMR experiments indicate that no significant global structural change occurs upon F228A mutation (Fig. S5), and hence, subtle differences in structure or position of the side chains around F228 appear sufficient to block dsRNA binding.

### Isothermal Titration Calorimetry

Interestingly, although the monomeric EBOV VP35 RBD is observed to both backbone-bind and end-cap dsRNA in the EBOV crystal structure, the monomeric MARV VP35 RBD is only observed to backbone-bind in the crystal structure presented here. We wondered if MARV VP35 is also able to end-cap in solution, but that end-capping was precluded by this particular crystal packing arrangement, or if MARV VP35 does not end-cap at all. We conducted further experiments to gain insights into the ability of MARV VP35 to end-cap dsRNA.

Control ITC experiments show that binding of MARV VP35 to single-stranded RNA (as opposed to dsRNA) is negligible, and also, that no heat is produced upon simple dilution of an 18-bp dsRNA into a buffer identical to that in which the VP35 RBDs was stored (Fig. S6). These control experiments indicate that any heat generated during the mixing of VP35 RBD with dsRNA results from a direct interaction between the two molecules.

**Figure 5:**
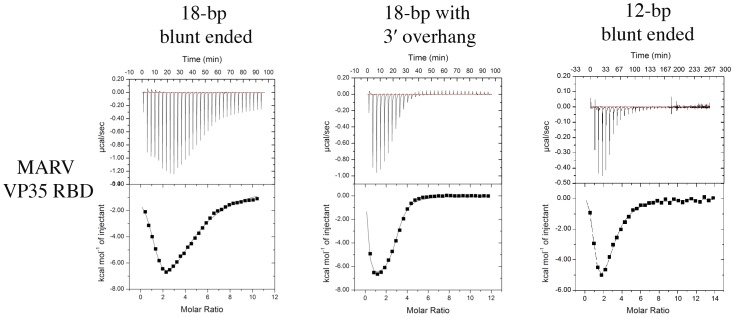
Isothermal calorimetry. Shown are raw data and isotherms for binding of MARV VP35 RBD to each of 18-bp blunt-ended dsRNA, 18-bp dsRNA with 3′ overhang and 12-bp blunt-ended dsRNA.

By isothermal calorimetry, MARV VP35 RBD interacts with blunt-ended, 18-bp dsRNA via a complex thermodynamic profile. Two major, distinct types of binding events take place that each possess a specific thermodynamic signature. Analysis of the data via a two-set-of-sites model indicates that both of the binding events observed (A and B) are energetically favorable and have binding affinities in the µM range. From the thermodynamic values derived from a two-set-of-sites model, it appears that binding event A is endothermic and driven by entropy, whereas binding event B is exothermic and driven by enthalpy. Binding event A has a stoichiometry of ∼1 MARV VP35 RBD binding to one 18-bp dsRNA, whereas binding event B has a stoichiometry of 2–3 MARV VP35 RBDs binding to each 18-bp dsRNA (Fig. 5 and Table 4).

**Table 4:** Thermodynamic parameters of binding determined by isothermal titration calorimetry (ITC) for dsRNA-binding domains of MARV VP35 with different dsRNA molecules.

		ΔG^A^ * (kcal/mol)	N^A^	*K_d_* ^A^ (µM)	ΔG^B^ (kcal/mol)	N^B^	*K_d_* ^B^ (µM)	N^(A+B)^
MARV VP35	18-bp dsRNA	−7.8	1.1	1.9	−6.2	2.6	28.6$	3.7
	18+3′ bp dsRNA	—&	0.1	—&	−7.5	3.1	3.4	3.2
	12-bp dsRNA	−8.6	1.0	0.5	−6.8	1.8	9.9	2.8

All reported values represent a calculated average determined from at least two independent measurements. Unless otherwise noted, the estimated error from replicate measurements is approximately 10% of the reported average values. Slight errors on the absolute stoichiometry values are also associated with errors in protein concentration measurements, as well as sample impurity. Superscripts A and B refer to events A and B wherever applicable. N represents number of molecules binding in that event (stoichiometry of binding).

* The change in Gibbs free energy (ΔG) was determined using the equation: ΔG_binding_ = RTlnK_d_ [47].

& There are too few measured points to confidently determine the ΔG_binding_ or the K_d_ of the first binding event (stoichiometry below 0.2).

$ The standard deviation for this binding event derived from four independent measurements (N^B^±0.3 and K_d_^B^±16.5) is significantly higher than the estimated error associated with other measurements.

In order to reveal the identity of the two events, the RBD was titrated into solutions of 3′ overhang-containing 18-bp dsRNA, blunt-ended 18-bp dsRNA, or blunt-ended 12-bp dsRNA. When the MARV VP35 RBD is titrated into 3′ overhang dsRNA instead of blunt-ended dsRNA, binding event A almost completely disappears. Using a two-set-of-binding-sites model to fit this data, the stoichiometry of binding event A to 3′-overhang dsRNA is 0.1∶1 (vs. 1.1∶1 for blunt-ended dsRNA). The stoichiometry of binding event B remains the same within experimental error (∼3.4∶1 for 3′ overhang dsRNA vs ∼2.6∶1 for blunt-ended dsRNA). When binding of the shorter, blunt-ended 12-bp oligo is compared to binding of the longer, blunt-ended 18-bp oligo, we note no changes to the binding event A within experimental error (1.0 vs 1.1 for 12-bp vs. 18-bp dsRNA). However, we do observe that in binding event B, fewer MARV VP35 RBDs bind to the shorter 12-bp dsRNA than to the longer 18-bp dsRNA. Specifically, ∼1–2 MARV VP35 RBDs are observed to bind 12-bp dsRNA while ∼2–3 bind 18-bp dsRNA.

The endothermic binding event A likely represents end-capping because it is blocked by the presence of a 3′ overhang at the end of the dsRNA and is unchanged by the length of the dsRNA. In addition, binding event A is seen to be driven by entropic effects, which are generally associated with the interaction of hydrophobic patches. Indeed, in the ebolavirus VP35 RBD-dsRNA crystal structures, end-capping is mostly mediated by hydrophobic residues. On the other hand, the exothermic binding event B likely represents backbone-binding because it is unaffected by the presence of a 3′ overhang and because more molecules can bind as the length of the oligo is increased. Hence, ITC suggests that an end-capping interaction does occur for MARV VP35 RBD in solution, even though it was not observed in this particular crystal packing arrangement.

### IFN antagonism by VP35 RBDs

**Figure 6:**
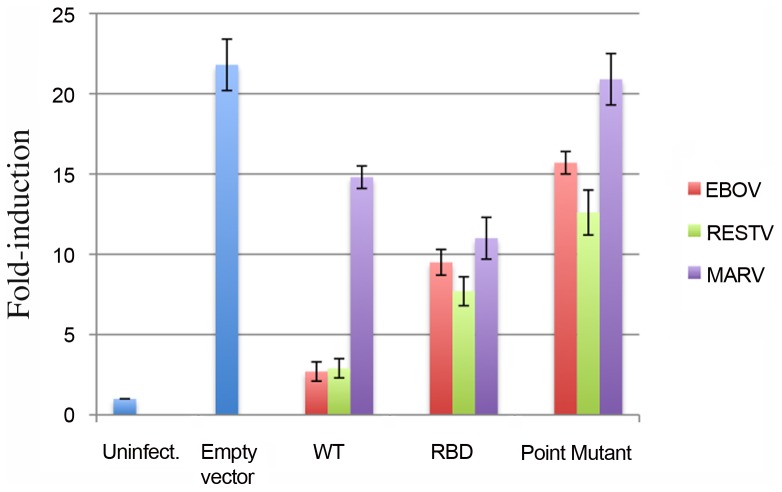
IFN antagonism by EBOV, MARV and RESTV VP35. HEK-293T cells were infected with Sendai virus and IFNβ promoter activity was determined by firefly luciferase expression driven by the human IFNβ promoter sequence in the presence of various versions of VP35. Uninfected cells and infected cells transfected with an empty vector were used as controls (blue bars). The inability of the RBD to reconstitute full inhibition of IFN signaling is the reason we call this region comprising residues 205–329 (and equivalent in EBOV) the RNA-binding domain (RBD) rather than the IFN inhibitory domain.

We compared the abilities of full-length VP35 and the VP35 RBD of MARV, EBOV and RESTV to antagonize activation of the IFNβ promoter in a firefly luciferase reporter assay. Negative controls for the IFNβ assay included R301A point mutations in MARV and RESTV and the equivalent R312A point mutation in EBOV. These point mutations knock out dsRNA binding, and it has been previously shown for EBOV, that loss of dsRNA binding by R312A diminishes IFN antagonism [10]. In the assay reported here, full-length MARV VP35 is ∼5 fold less effective in inhibition of the human IFNβ promoter than the full-length VP35s of EBOV or RESTV (Fig. 6). Interestingly, the MARV VP35 RBD is slightly more effective as an IFN antagonist than the full-length protein. By contrast, the VP35 RBDs of EBOV and RESTV are ∼4 and ∼2-fold less effective than their full-length proteins. For all three proteins, the point mutation R301A in RESTV and MARV and its equivalent R312A in EBOV are detrimental to IFN antagonism. However, the mutations in EBOV and RESTV VP35 still result in some weak level of IFN antagonism (Fig. 6).

## Discussion

VP35 plays multiple roles in the viral life cycle. One of these roles, IFN antagonism, involves its C-terminal dsRNA-binding domain. Previous crystal structures of the VP35 RBDs from RESTV and EBOV, in complex with blunt-ended dsRNAs, have revealed that two molecules of VP35 RBD bind to the end of the dsRNA, each molecule by a different mechanism [10], [22]. The first, “backbone-binding” VP35 interacts solely with the backbone of dsRNA using the residues in the central basic patch. The second, “end-capping” VP35 packs against the terminus of dsRNA using residues in a hydrophobic patch, and also interacts with a portion of end-proximal dsRNA backbone using residues in the central basic patch. Together the backbone-binding and the end-capping VP35s form an asymmetric dimer held together by a network of hydrogen bonds.

The crystal structure of MARV VP35 RBD alone and in complex with a 12-bp palinodromic dsRNA shows that the individual dsRNA oligonucleotides have self-assembled into an essentially continuous dsRNA that is completely coated by copies of the VP35 RBD with no significant structural changes in the RBD upon binding dsRNA. No end-capping interactions are observed. This structure illustrates an arrangement by which a filovirus VP35 RBD could indeed coat the dsRNA backbone between the ends to block backbone-sensitive immune surveillance molecules like RIG-I and MDA5.

**Figure 7:**
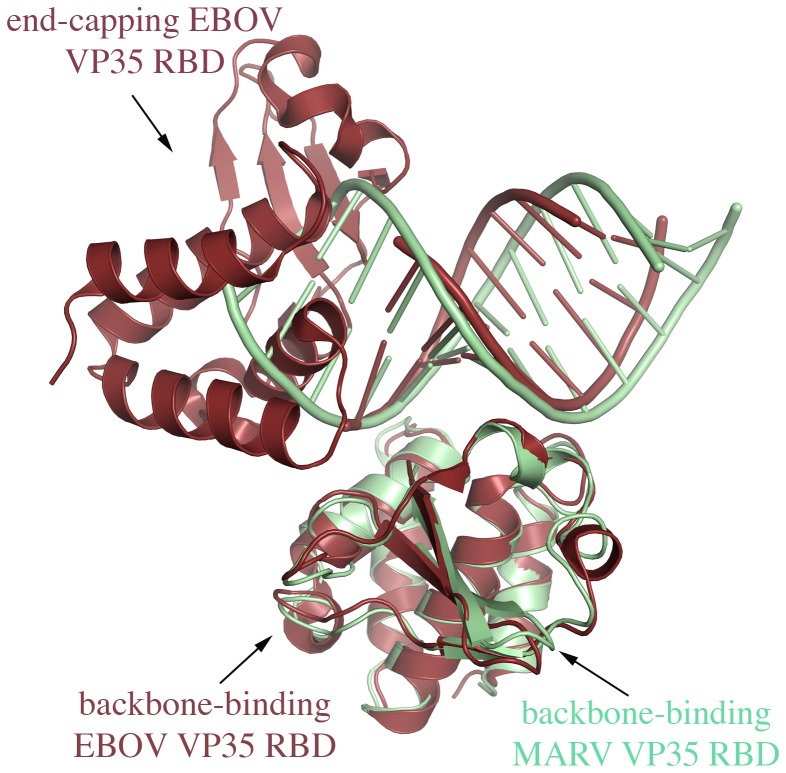
Coating and end-capping of filovirus VP35 RBDs. The MARV-dsRNA crystal structure (green) is superimposed with that of EBOV (brick red). In the MARV crystals, all copies of VP35 in the asymmetric unit exhibit backbone binding, while in the EBOV crystals, at each end of the dsRNA, one VP35 binds the backbone while the other caps the end. Any of the four copies of MARV VP35 in the asymmetric unit superimpose with the backbone-binding VP35s of EBOV. Residues in MARV equivalent to those of the EBOV backbone-binding VP35 that form the dimer interface with the EBOV end-capping VP35, do not interact with another MARV VP35, but instead interact with the dsRNA backbone. Biophysical experiments indicate that in solution, MARV VP35 can both backbone-bind and end-cap, so the differences depicted here may be differences between complexes captured in different crystal packing arrangements, rather than inherent differences between MARV and EBOV.

The MARV VP35 RBD – dsRNA interaction is similar to that of the backbone-binding VP35 RBDs of EBOV and RESTV in complex with dsRNA (Fig. 7). However, some differences between MARV and EBOV/RESTV occur in individual residues of the central basic patches of the different viruses. Dot-blot binding analysis reveals that MARV R301, K298 and R271 are critical to binding dsRNA. By contrast, K298 in RESTV and its equivalent K309 in EBOV, are not critical for dsRNA binding [10]. In MARV, the residues K311, K328 and I329 are not critical for dsRNA binding (Table 5). By contrast in EBOV, residues R322 and K339 are critical for dsRNA binding [10].

**Table 5:** Importance of individual residues in the central basic patch of filovirus VP35 RBDs for binding dsRNA [10], [22].

Residue in EBOV	dsRNA binding	Residue in RESTV	dsRNA binding	Residue in MARV	dsRNA binding
K282	ND	K271	+	R271	−
R305	+++	R294	+++	R294	ND
K309	++	K298	++	K298	−
R312	−	R301	−	R301	−
R322	−	R311	ND	K311	−
K339	−	K328	−	K328	+++
wild-type	+++	wild-type	+++	wild-type	+++

Of additional interest is position F228 in MARV (F239 in EBOV). F228 forms an aromatic contact with the final dsRNA base in the end-capping VP35s in both EBOV and RESTV structures, but makes no interaction with dsRNA in the backbone-binding VP35s in EBOV, RESTV or MARV. We wondered if F228 is instead important for maintenance of the VP35 structure, rather than formation of a specific dsRNA contact. A leucine at this position may be able to maintain the structural integrity of the hydrophobic pocket, yet would not be able to form the same aromatic base-stacking interactions with the 3′ dsRNA base.

Indeed, dot-blot experiments indicate that in MARV, F228A abrogates dsRNA binding, but that the alternate mutation, F228L, retains wild-type levels of dsRNA binding. Similarly, H240 in EBOV (equivalent to H229 in MARV) is located in the hydrophobic pocket adjacent to the phenylalanine. This histidine does not contact dsRNA in any of the structures determined, yet its mutation abrogates dsRNA binding [10]. Although no major structural rearrangement occur with an F228A mutation (Fig. S5), mutation within this hydrophobic pocket may cause subtle conformational changes or residue re-positioning that are nonetheless sufficient to abrogate dsRNA binding.

Our isothermal calorimetry experiments indicate that in solution, as well as in crystals, MARV VP35 RBDs can coat the dsRNA sugar-phosphate backbone between the ends. Although end-capping also occurs in solution, it was not observed in crystals and does not seem to be a requirement of the MARV VP35 RBD to bind dsRNA.

What would MARV VP35 end-capping look like? In infected cells, we would expect that MARV VP35 RBD could continuously coat any exposed backbone of dsRNA. At the terminus of the dsRNA, two VP35 RBD molecules likely collapse to cap the end. At this end cap, the residues in the partially exposed central basic patch of the final backbone-binding RBD could be neutralized by extensive hydrogen bonding with acidic residues from the end capping subunit stabilizing the end cap assembly (Fig. 7). The favorable interactions of these otherwise unsatisfied residues could form an asymmetric dimer as observed in both EBOV and RESTV structures.

Recognition of dsRNA, a unique product of viral infection by host proteins RIG-I and MDA5 leads to a potent immune response involving production of type I IFNs and proinflammatory cytokines [30]–[32]. Recent crystal structures of RIG-I in complex with dsRNA reveal that the C-terminal repressor domain caps the hydrophobic terminus and 5′-ppp of dsRNA, while the helicase domains of RIG-I bind the backbone of dsRNA [25], [26], [33]. Further, both RIG-I and MDA5 have been shown to cooperatively oligomerize on dsRNA in a length-dependent fashion [28], [29], with stoichiometry of binding dependent on the length of the dsRNA [34]. These recent findings suggest that evading host detection of viral dsRNA could be best achieved if VP35 can both coat the backbone and cap the ends of dsRNA. The results presented here indicate that MARV VP35 can indeed, both cap the ends and coat the expanses of dsRNA backbone between the ends.

The critical question of how full-length VP35 interacts with dsRNA is still to be answered. Full-length VP35 from all the filoviruses oligomerizes via an N-terminal coiled-coil domain [21], [35], [36]. It is unclear if the individual RBDs interact with each other as observed in EBOV and RESTV crystal structures, or if they exist as independent monomers as seen in the MARV crystal structure. Our IFN suppression assay suggests that for MARV, the RBD confers IFN suppression that is similar to that conferred by the full-length VP35 oligomer. By contrast, the EBOV or RESTV RBDs alone confer IFN suppression that is weaker than that achieved by the full-length protein. These results suggest that in the ebolaviruses, the rest of the VP35 molecule controls oligomerization in some way that does not occur for MARV, or that the rest of the ebolavirus VP35 contributes to IFN inhibition in some other way that does not occur for MARV.

Indeed, the extent of IFN antagonism achieved by MARV VP35 is lower than that achieved by the ebolaviruses. This difference is possibly due to the 3–5 fold lower affinity of MARV VP35 for dsRNA. However, binding of dsRNA by VP35 does not appear to be the sole determinant of virulence, as RESTV is nonpathogenic to humans while recent outbreaks of MARV have occurred with up to 90% human lethality. Indeed, VP35 has other functions in the infected cell as well, numerous host and viral factors determine the kinetics of viral replication, and additional avenues of immunosuppression are facilitated by separate proteins in the different viruses. In the ebolaviruses, the VP24 protein contributes to immunosuppression, while in MARV, additional immunosuppressive functions are instead contributed by the VP40 protein [37], [38].

In summary, the crystal structures, biochemical and biophysical experiments reported here illustrate coating of the dsRNA backbone as a likely mechanism of IFN antagonism for MARV. This mode of dsRNA recognition is in contrast to the end-only recognition observed in crystal structures of VP35 RBD from the ebolaviruses. This additional mode of dsRNA recognition by backbone coating explains how MARV can avoid length-dependent sensing of its dsRNA, and provides an alternative template for design of antiviral therapeutics.

## Materials and Methods

### Plasmids, mutagenesis and RNA

The construct for expression of MARV VP35 RBD contains residues 205–329 and was subcloned into a pET46b vector. This construct is similar in length to that crystallized for EBOV. A slightly shorter construct of MARV VP35 comprising residues 205–327 was made using a pET-46 Ek/LIC vector kit. Site-directed mutagenesis using a Phusion Site-directed mutagenesis kit was employed to introduce point mutations in the clones.

Synthetic RNA oligomers were obtained from Integrated DNA Technologies. The sequences of RNA oligos used are as follows: (for crystallization) 12-bp palindrome 5′ CUA GAC GUC UAG 3′; (for dot blot and ITC experiments) 18-bp blunt ended sense 5′ AGA AGG AGG GAG GGA GGA 3′; 18-bp blunt ended anti-sense 5′ UCC UCC CUC CCU CCU UCU 3′; 18-bp with 3′ overhang sense 5′ AGA AGG AGG GAG GGA GGA GAG 3′; 18-bp with 3′ overhang anti-sense 5′ CUC UCC UCC CUC CCU CCU UCU 3′; 18-bp with 5′ overhang sense 5′ GAG AGA AGG AGG GAG GGA GGA 3′; 18-bp with 5′ overhang anti-sense 5′ UCC UCC CUC CCU CCU UCU CUC 3′; 12-bp blunt ended sense 5′ GAC ACC UGA UUC 3′; 12-bp blunt ended anti-sense 5′ GAA UCA GGU GUC 3′.

### Protein expression and purification

The protein encoding the MARV VP35 RBD was expressed in *E. coli* R2 cells. The cells were grown in a 50 mL overnight culture supplemented with ampicillin and chloromphenicol at 37°C with shaking at 300 rpm. The overnight culture was introduced into 1 L LB broth media supplemented with ampicillin and grown to an OD_600 nm_ of 0.6 and induced with 1.0 mM IPTG. The protein was expressed over 5 h with shaking at 37°C. The cells were harvested by centrifugation and lysed using a sonicator in a wash buffer containing 20 mM Tris, pH 7.5, 50 mM NaCl and 10 mM imidazole. The lysate was separated from the cell debris by centrifugation at 16,000 rpm and applied to a His-Trap column (GE healthcare) pre-equilibrated with wash buffer. The column was washed with 10 column volumes of wash buffer, followed by another wash, with wash buffer containing 30 mM imidazole. The protein was eluted in wash buffer containing 300 mM imidazole. The 6x-His Tag was cleaved by incubating the protein with Tobacco Etch Virus protease overnight in buffer containing 25 mM Bis Tris pH 6.5, 50 mM NaCl, 5 mM DTT. The protein was further purified using ion exchange chromatography. A Mono S column was equilibrated with buffer containing 25 mM Tris, pH 7.5, 50 mM NaCl, 5 mM TCEP and the protein was eluted with a gradient of NaCl. The protein fractions were further purified and buffer exchanged into 10 mM Tris, pH 8.0, 200 mM NaCl, 2 mM TCEP by Superdex 75 size exclusion. The shorter construct of MARV VP35 RBD (construct containing residues 205–327) and mutants were expressed and purified in a similar fashion. EBOV and RESTV VP35 RBDs used in RNA binding assays were expressed and purified from constructs containing residues 216–340 and 205–329, respectively, similar to MARV VP35 RBD.

### Crystallization and structure determination

The MARV VP35 RBD was incubated in 1∶1 ratio with various crystallization solutions from sparse matrix screens in a hanging drop format. The protein crystallized as clusters of needles in 2–2.4 M ammonium sulfate, 100 mM sodium acetate, pH 4.6. The individual needles were harvested and cryoprotected in sequential soaks of well solution containing 5%, 10%, 15% and 20% glycerol and were flash cooled under liquid nitrogen for diffraction experiments.

For crystallization of the MARV VP35 RBD-dsRNA complex, MARV VP35 RBD was incubated with 12-bp dsRNA in a RNA∶protein molar ratio of 1∶1.2 for 2 h. An initial crystallization hit of the complex was obtained in the PEG/Ion sparse matrix screen (Hampton Research) condition 0.1 M Bis-Tris, pH 6.5, 2% v/v Tacsimate, pH 6.0, and 20% PEG 3350 using sitting drop vapor diffusion. Needle-like crystals of dimensions 200 µm×30 µm×30 µm were obtained after optimization in condition 0.1 M Bis-Tris, pH 6.2, 2% v/v Tacsimate, pH 7.0, and 18% PEG 3350 and grew over a period of 3–4 days. The crystals were cryoprotected in well solution supplemented with 15% glycerol prior to flash cooling in liquid nitrogen for diffraction experiments.

Data for the uncomplexed MARV VP35 RBD were collected on beamline 8.3.1 of the ALS (Advanced Light Source Berkeley, CA). Data for the MARV VP35 RBD – dsRNA complex were collected on beamline 5.0.2 of the ALS. For MARV VP35 RBD, data were collected over a rotation range of 180° with an oscillation range of 1° and 4 s exposure per frame, at a crystal-to-detector distance of 200 mm. For MARV VP35 RBD-dsRNA complex, data were collected over a rotation range of 180° with an oscillation range of 1° and 1 s exposure per frame, at a crystal-to-detector distance of 350 mm. The data were integrated and scaled using the programs D*TREK and HKL2000 for the unbound MARVP35 RBD and the dsRNA complex, respectively [39], [40]. Data collection statistics are summarized in Table 1.

The structure of the unbound MARV VP35 RBD was determined by molecular replacement with the structure of the RESTV VP35 RBD (PDB code 3KS4) as the search model using the program Phaser in the Phenix suite [41], [42]. The asymmetric unit contains one molecule of the MARV VP35 RBD. The structure of MARV VP35 bound to 12-bp RNA was determined by molecular replacement with the structure of the MARV VP35 RBD as the search model, using the program Phaser. The initial model from molecular replacement contained four copies of the MARV VP35 RBD in the asymmetric unit. All the models were refined using Refmac and Phenix, followed by model building using the program Coot [43]–[45]. Initial difference Fourier maps clearly showed positive density for the bound 12-bp dsRNA in the complex structure as well as water molecules that were subsequently built into the model. The final refinement statistics are shown in Table 2.

### Dot blot experiments for RNA binding

The 5′ end of the sense strand was labeled with ^32^P using γ-^32^P-ATP and T4 Polynucleotide kinase (New England Bio Labs). The labeled oligo was duplexed using a 1.5 molar excess of the anti-sense strand by heating for 5 min at 90°C in 10 mM Tris, pH 8.0, 200 mM NaCl and 2 mM TCEP (binding buffer) followed by slow cooling down to room temperature. Trace amounts of RNA were incubated with increasing amounts of protein (in binding buffer) in a volume of 50 µl for 2 h [46]. The reaction mixture was pulled through successive layers of protein-binding membrane (Protran-BA85 from Whatman) and RNA-binding membrane (Hybond-N+ from Amersham) using a Minifold I Dot-Blot system – 96 well apparatus under vacuum. The membranes were separated, dried in air for 10 min, wrapped in transparent plastic wrap and exposed overnight to a phosphor screen (Amersham). The screen was imaged using a Typhoon phosphorimager and the images were quantified using Imagequant software (GE Healthcare). The ratio of RNA bound to protein versus RNA bound to the membrane was obtained and the data were fit to one-site specific binding Hill equation using GraphPad Prism software version 5.0d (www.graphpad.com). Binding experiments were done at least in duplicate.

### Isothermal Titration Calorimetry

Isothermal titration calorimetry (ITC) experiments were carried out using a MicroCal iTC200 instrument (GE). Extensive dialysis of all proteins and RNA molecules against 10 mM Tris, pH 8.0, 200 mM NaCl, 2 mM TCEP buffer was performed prior to ITC experiments. Protein and RNA concentrations were determined by UV absorbance using calculated extinction coefficients (ProtParam, Gasteiger, 2005). All ITC experiments were carried out in duplicate. MARV VP35 RBD was in the syringe at concentrations ranging between 1–2 mM, while the RNA molecules were in the cell at concentrations ranging between 15–40 µM. One experiment consisted of either 16 injections of 2.5 µl each or 32 injections of 1.25 µl each with injection interval of 180 s and reference power of 5 µcals. For the experiment involving 12-bp dsRNA, duplicate experiments were also done with time intervals of 300 s and 500 s between injections to allow the system to reach equilibrium. Affinity constants (K_d_) and molar reaction enthalpy (ΔH) were calculated by fitting the integrated titration peaks with Origin 7.0 software using a “two-sets-of-sites” binding model, as deemed appropriate by visual inspection of the raw data. The first data point corresponding to a 0.5 µl injection was discarded in the analysis of all the experiments. The change in Gibbs free energy, ΔG, was then calculated as ΔG_binding_ = RTlnK_d_ [47].

### IFN antagonism reporter assay

To construct the IFNβ promoter firefly luciferase reporter plasmid, the human IFNβ promoter sequence was amplified from human genomic DNA using Phusion DNA polymerase (New England Biolabs) and the following primers: IFNβ −125 (5′-CAG GGT ACC GAG TTT TAG AAA CTA CTA AAA TG-3′) and IFNβ +34 (5′-GTA CTC GAG CAA AGG CTT CGA AAG G-3′). The primers correspond to the human IFNβ promoter sequence from −125 to +34 relative to the transcription start site (+1). The PCR fragment was digested with the restriction enzymes *KpnI* and *XhoI* and then ligated into a similarly digested pGL4.10(luc2) vector (Promega).

HEK-293T cells were grown in complete medium (Dulbecco's modified Eagle's medium supplemented with 10% fetal bovine serum) and plated in 24-well poly-D-lysine coated plates at 95,000 cells per well. Using LT-1 (Mirus), cells were transfected with 0.5 µg of the IFNβ promoter reporter plasmid (pGL-IFNβ luc) and 1.0 µg of the indicated pCAGGS VP35 protein expression plasmids or empty pCAGGS plasmid. The full-length VP35 constructs comprise residues 1–329 for MARV and RESTV and residues 1–340 for EBOV. The VP35 RBD constructs comprise residues 205–329 for MARV and RESTV and residues 216–340 for EBOV. Twenty-four h after transfection, cells were infected with Sendai virus for 1 h at a multiplicity of infection of 5. Cells were washed three times after infection and complete medium was replaced onto cells. Twenty-four h after infection, cells were lysed with Glo Lysis Buffer (Promega) and firefly luciferase expression was determined using Steady-Glo Assay Buffer (Promega). Data were obtained from three independent experiments carried out in triplicate. The relative fold increase of firefly luciferase expression was determined by comparing Sendai virus infected cells to uninfected control cells.

### NMR analysis

The protein samples were dialyzed overnight into 10 mM Tris, pH 6.5, 200 mM NaCl, 5 mM TCEP and concentrated to a final concentration of ∼380 µM for the MARV VP35 RBD wild type protein and ∼1.2 mM for the F228A mutant. D_2_O was added to each sample to 5% v/v. A homonuclear 2D NOESY with a mixing time of 100 ms was obtained at 298K using a Bruker DRX600 MHz using a 5 mm TXI cryo-probe with Z-gradient. The data matrix consisted of 8192 complex points in t1 and 1024 complex points in t2. The data was processed using NMRPipe [48] and analyzed using NMRView [49].

## Supporting Information

Figure S1 **The conserved first basic patch in MARV VP35 RBD.** (A) Cartoon representation of MARV VP35 RBD. Conserved residues are shown in ball and stick and colored orange. (B) Electrostatic surface representation of the RBD showing the basic patch with a limit of ±3 *k_B_*T/e_c_.
(TIF)

Figure S2 **The MARV VP35 RBD interface between adjacent monomers (A – shown in green, B-shown in yellow).** This interaction buries a surface area of 340 Å^2^. A hydrogen bond between A302 and T219 (residues represented as sticks) is shown as a black dashed line.
(TIF)

Figure S3 **Dot blot binding assay of MARV VP35 RBD containing an F228L point mutation (circles), or a shorter version of the RBD from which the final two residues were deleted (now containing residues 205–327; squares) compared to wild-type (triangles), binding to 18-bp blunt-ended dsRNA.**
(TIF)

Figure S4 **Dot blot binding assay of (A) EBOV VP35 RBD and (B) RESTV VP35 RBD binding to 18-bp blunt ended dsRNA.** (C) MARV, EBOV and RESTV VP35 RBD binding to 18-bp dsRNA with 3′ overhang. The K_d_ and Hill coefficients of binding are shown in Table 3.
(TIF)

Figure S5 **2D-NOESY spectrum comparing wild-type MARV VP35 RBD (shown in black) to the F228 mutant (shown in red).** The overlay of the spectrum suggests that no global conformational changes occur upon the F228A mutation.
(TIF)

Figure S6 **Control ITC binding isotherms for (A) Buffer dilution to 18-bp dsRNA; (B) MARV VP35 RBD titrated into 18-bp single stranded RNA.**
(TIF)
