# Supplementary figures and images for: Marburg Virus VP35 Can Both Fully Coat the Backbone and Cap the Ends of dsRNA for Interferon Antagonism
Source: PLoS Pathog. 2012 Sep 13;8(9):e1002916. doi: 10.1371/journal.ppat.1002916 (PMC3441732; doi:10.1371/journal.ppat.1002916)

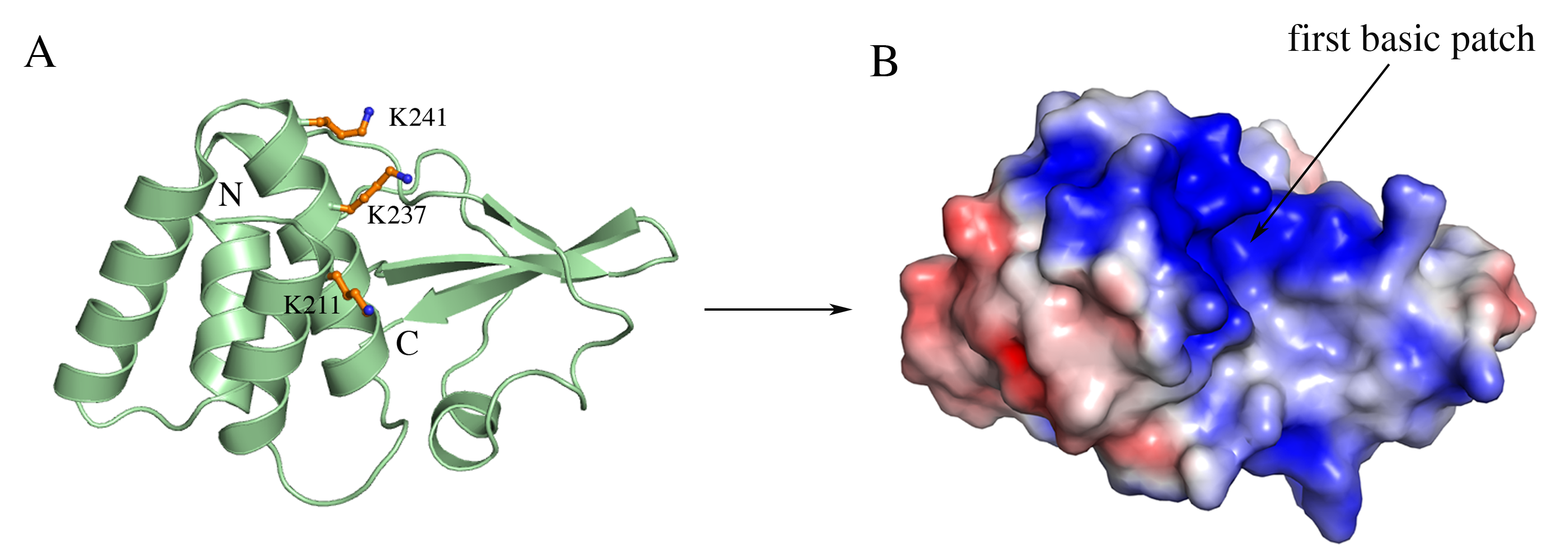

Supplement: Figure S1 — The conserved first basic patch in MARV VP35 RBD. (A) Cartoon representation of MARV VP35 RBD. Conserved residues are shown in ball and stick and colored orange. (B) Electrostatic surface representation of the RBD showing the basic patch with a limit of ±3 kBT/ec. (TIF) [file ppat.1002916.s001.tif]

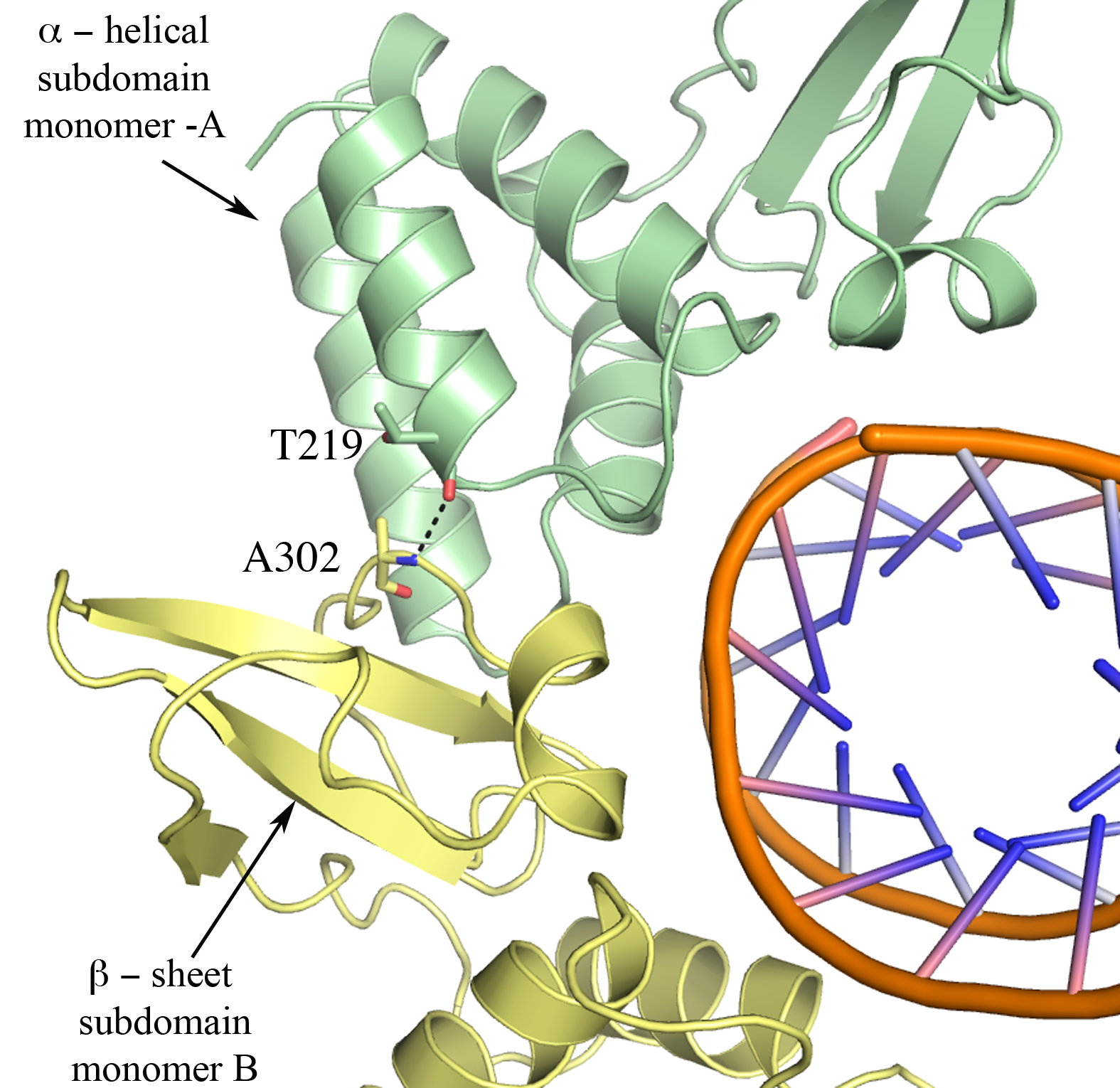

Supplement: Figure S2 — The MARV VP35 RBD interface between adjacent monomers (A – shown in green, B-shown in yellow). This interaction buries a surface area of 340 Å2. A hydrogen bond between A302 and T219 (residues represented as sticks) is shown as a black dashed line. (TIF) [file ppat.1002916.s002.tif]

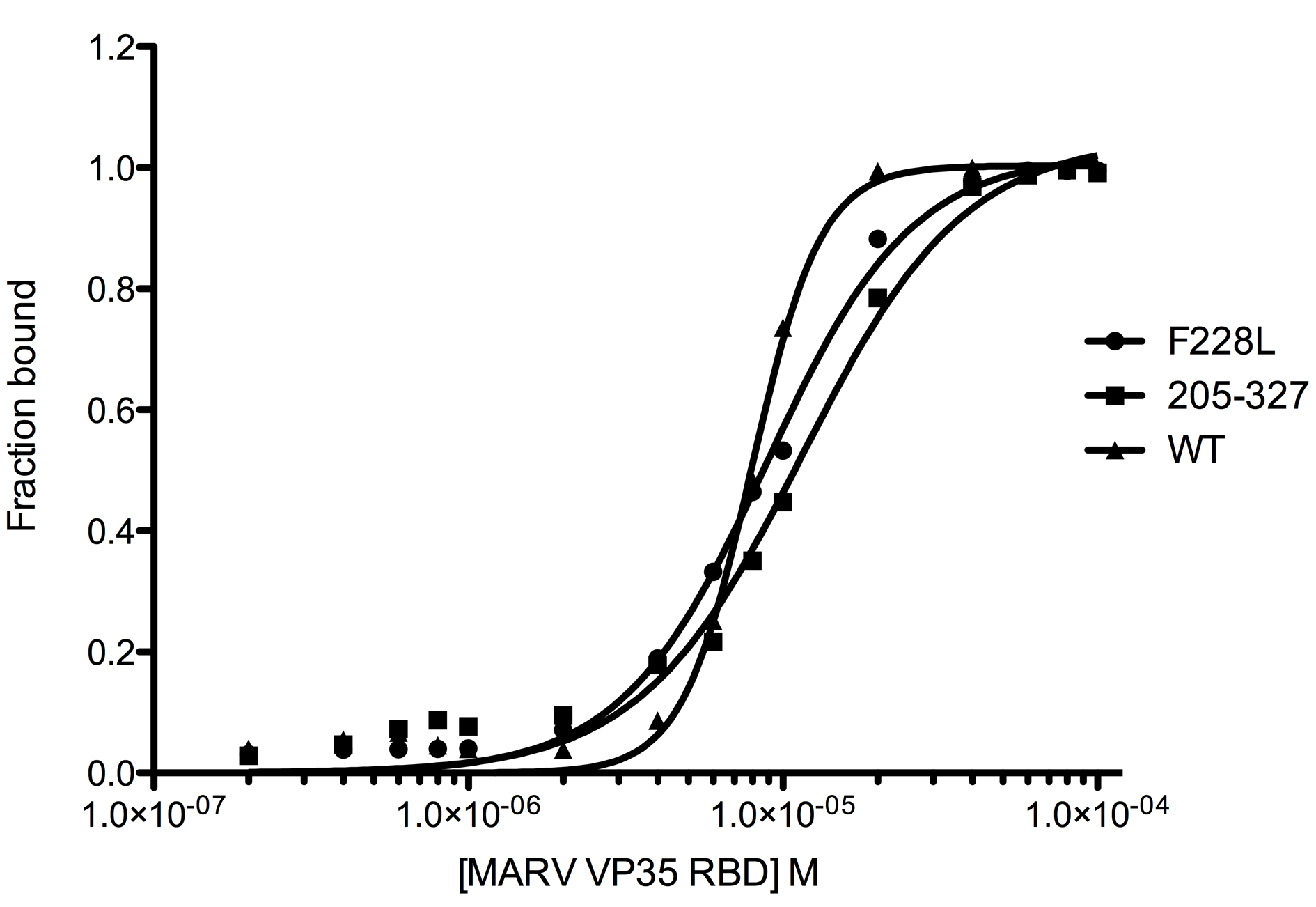

Supplement: Figure S3 — Dot blot binding assay of MARV VP35 RBD containing an F228L point mutation (circles), or a shorter version of the RBD from which the final two residues were deleted (now containing residues 205–327; squares) compared to wild-type (triangles), binding to 18-bp blunt-ended dsRNA. (TIF) [file ppat.1002916.s003.tif]

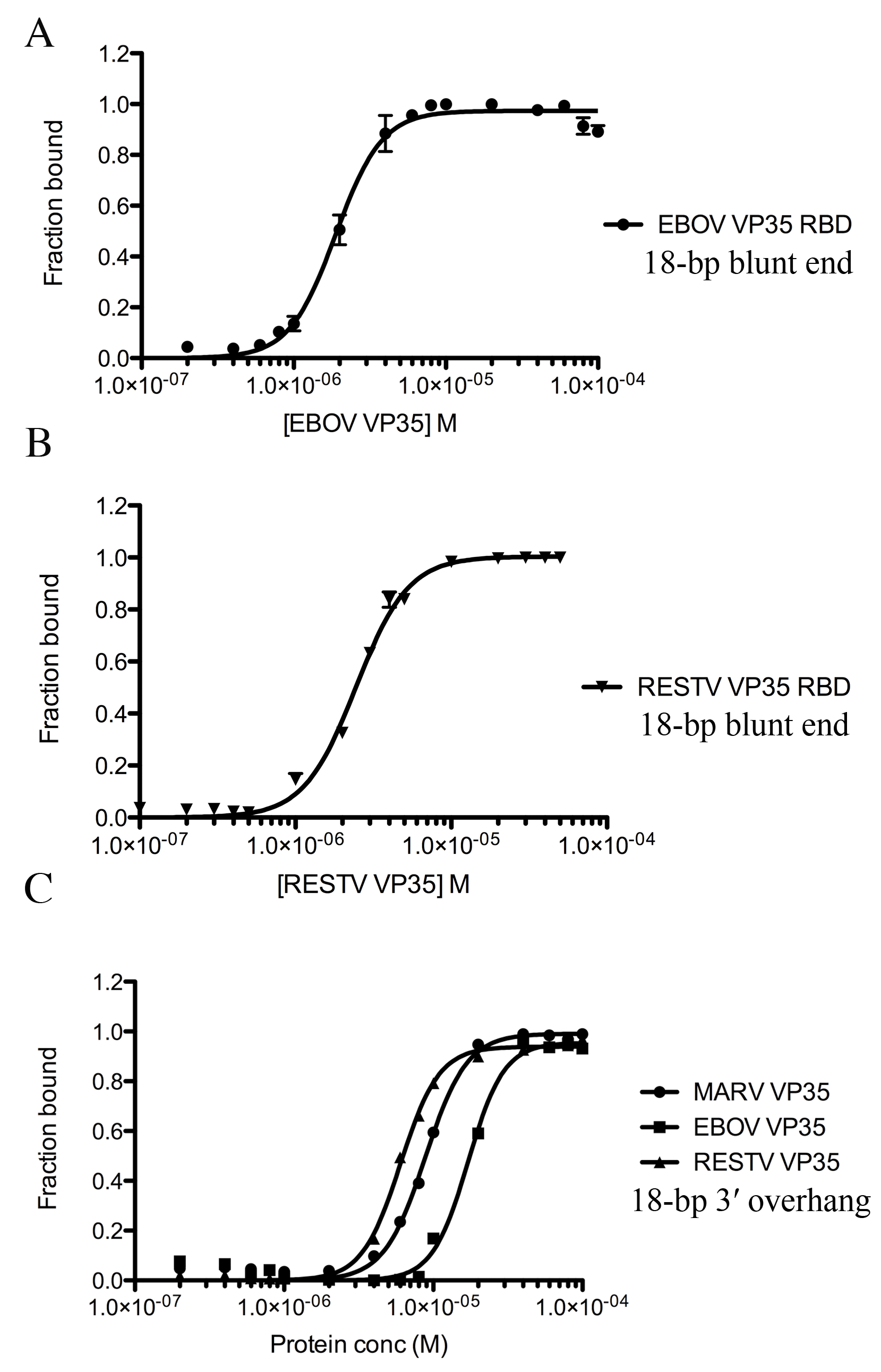

Supplement: Figure S4 — Dot blot binding assay of (A) EBOV VP35 RBD and (B) RESTV VP35 RBD binding to 18-bp blunt ended dsRNA. (C) MARV, EBOV and RESTV VP35 RBD binding to 18-bp dsRNA with 3′ overhang. The Kd and Hill coefficients of binding are shown in Table 3. (TIF) [file ppat.1002916.s004.tif]

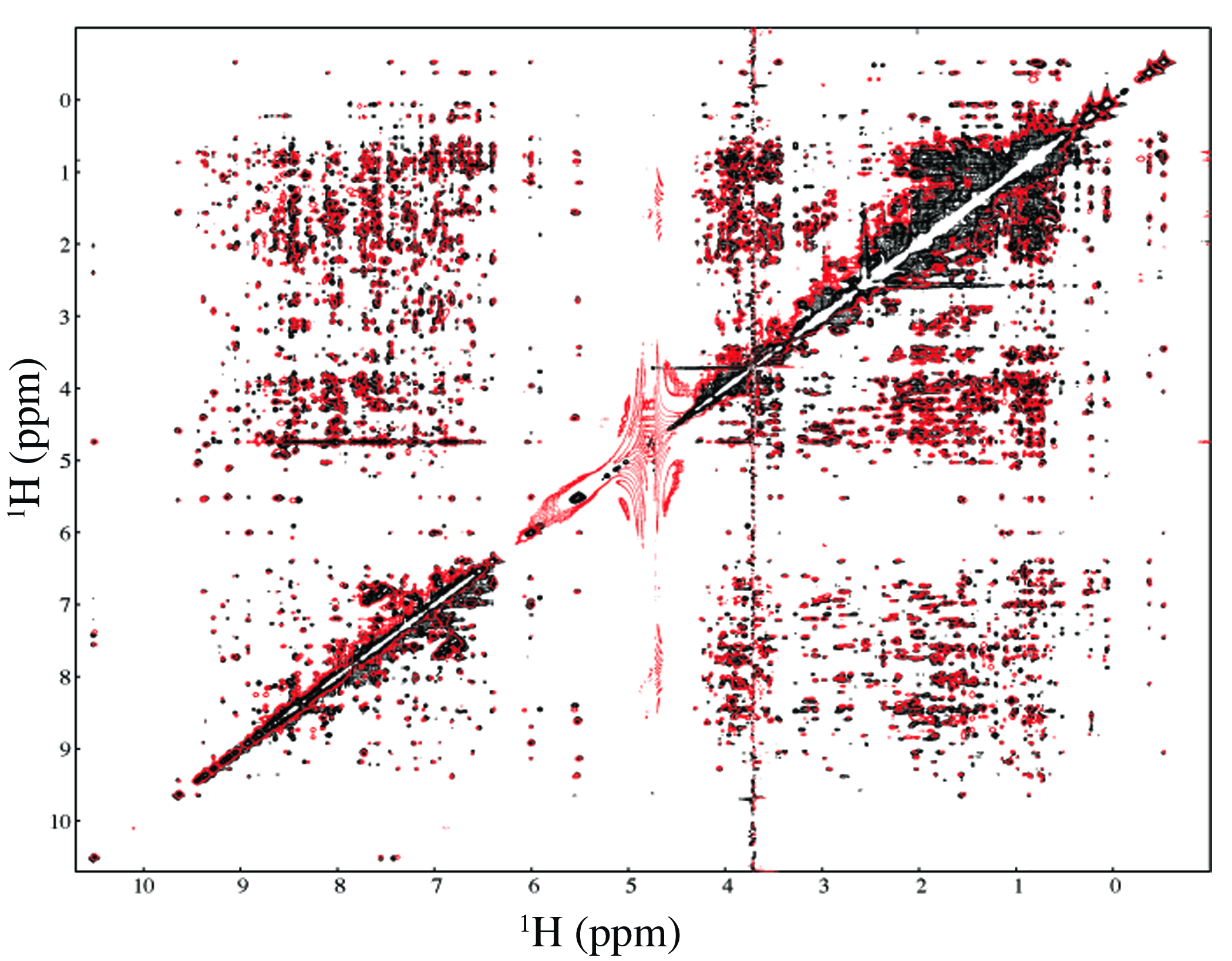

Supplement: Figure S5 — 2D-NOESY spectrum comparing wild-type MARV VP35 RBD (shown in black) to the F228 mutant (shown in red). The overlay of the spectrum suggests that no global conformational changes occur upon the F228A mutation. (TIF) [file ppat.1002916.s005.tif]

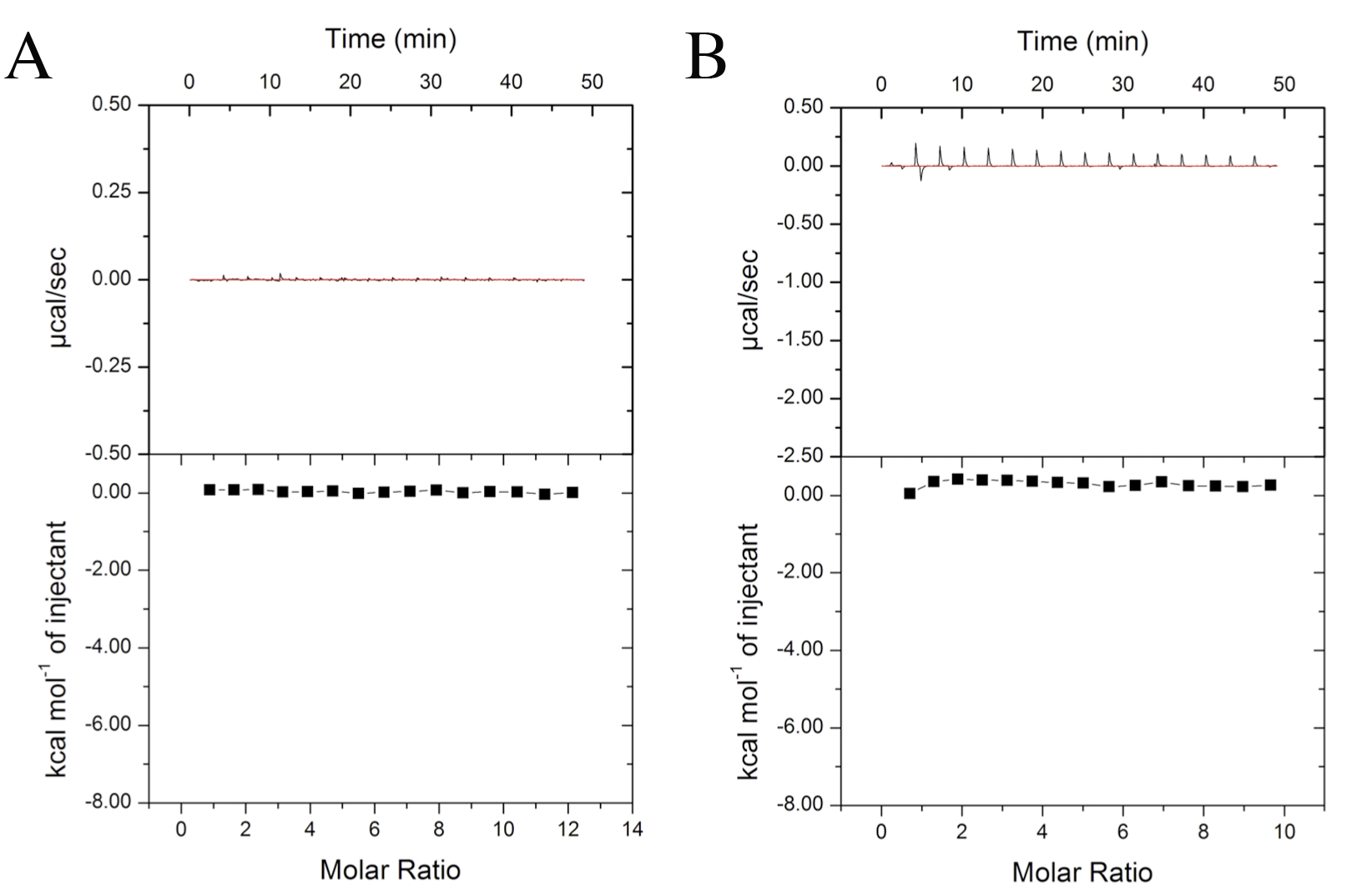

Supplement: Figure S6 — Control ITC binding isotherms for (A) Buffer dilution to 18-bp dsRNA; (B) MARV VP35 RBD titrated into 18-bp single stranded RNA. (TIF) [file ppat.1002916.s006.tif]
